# Supplementary material for: A stroke detection and discrimination framework using broadband microwave scattering on stochastic models with deep learning
Source: Sci Rep. 2021 Dec 20;11:24222. doi: 10.1038/s41598-021-03043-y (PMC8688451; doi:10.1038/s41598-021-03043-y)
Supplement: Supplementary file 1 — Supplementary Tables. [file 41598_2021_3043_MOESM1_ESM.pdf]

Supplemental Materials - A Stroke Detection and Discrimination Framework Using Broadband Microwave Scattering on Stochastic Models with Deep Learning

SM Table 1. Classification Network Architecture

Layer (type)	Output Shape	Param #
Layer_1 (Dense), sigmoid activation	(None, 1281)	3283203
Dropout_1 (7.5% Dropout)	(None, 1281)	0
Layer_2 (Dense) sigmoid activation	(None, 640)	820480
Dropout_2 (7.5% Dropout)	(None, 640)	0
Layer_3 (Dense), sigmoid activation	(None, 64)	41024
Dropout_3 (7.5% Dropout)	(None, 64)	0
Layer_4 (Dense) sigmoid activation	(None, 32)	2080
Dropout_4 (7.5% Dropout)	(None, 32)	0
Layer_5 (Dense) sigmoid activation	(None, 16)	528
Dropout_5 (7.5% Dropout)	(None, 16)	0
dense_1 (Dense), sigmoid activation	(None, 1)	17

Total params: 4,147,332
Trainable params: 4,147,332
Non-trainable params: 0

Hyperparameters:
Layer initialization: Truncated normal distribution (mean=0, sigma=0.75)
Loss = Binary cross entropy
Optimizer = Adam
Epochs = 30
Batch size = 100

SM Table 2. Discrimination Network Architecture

Layer (type)	Output Shape	Param #
Layer_1 (Dense), relu activation	(None, 15372)	39398436
Dropout_1 (7.5% Dropout)	(None, 15372)	0
Layer_2 (Dense), relu activation	(None, 2562)	39385626
Dropout_2 (7.5% Dropout)	(None, 2562)	0
Layer_3 (Dense), relu activation	(None, 1024)	2624512
Dropout_3 (7.5% Dropout)	(None, 1024)	0
Layer_4 (Dense), relu activation	(None, 1024)	1049600
Dropout_4 (7.5% Dropout)	(None, 1024)	0
dense (Dense), linear activation	(None, 4)	4100

Total params: 82,462,274
Trainable params: 82,462,274
Non-trainable params: 0

Hyperparameters:
Layer initialization: Truncated normal distribution (mean=0, sigma=0.005)
Loss = mean squared error
Optimizer = Adam
Epochs = 1500
Batch size = 80

Downloadable data:
https://www.dropbox.com/sh/krubtlhlgbm3pg0/AAD2iGi2LL2otPSm7FyweK_Ta?dl=0
